# The Effect of Tyre and Road Wear Particles on the Terrestrial Isopod *Armadillidium pallasii*

**DOI:** 10.3390/biom14121640

**Published:** 2024-12-20

**Authors:** Giorgia Torreggiani, Chiara Manfrin, Anita Giglio, Andrea Dissegna, Cinzia Chiandetti, Paola Giotta, Monia Renzi, Serena Anselmi, Tecla Bentivoglio, Agnieszka Babczyńska, Silvia Battistella, Paolo Edomi, Piero G. Giulianini

**Affiliations:** 1 Department of Life Sciences, University of Trieste, 34127 Trieste, Italycchiandetti@units.it (C.C.); edomi@units.it (P.E.); giulianini@units.it (P.G.G.); 2 Department of Biology, Ecology and Earth Science, University of Calabria, 87036 Rende, Italy; anita.giglio@unical.it; 3 Bioscience Research Center, 58015 Orbetello, Italy; 4 Faculty of Natural Sciences, University of Silesia in Katowice, 40-007 Katowice, Poland; agnieszka.babczynska@us.edu.pl

**Keywords:** behaviour, growth, habituation, immune responses, isopods, plastic particle pollution, stress, terrestrial woodlouse

## Abstract

(1) Car tyre microplastic particles (TMPs) significantly contribute to global microplastic pollution, with an estimated annual production of 6 million tonnes. However, the impact of TMPs, particularly tyre and road wear particles (TRWPs), resulting from tyre abrasion on the road on terrestrial organisms, is poorly understood. This study investigated the effects of TMPs and TRWPs on the growth, immune response, behaviour, and cognition of the woodlouse *Armadillidium pallasii* over 30 days; (2) TMPs and TRWPs were mixed together in the first experiment and provided at different concentrations of 1.25%, 2.5%, 5%, and 10% (*w*/*w*), and with soil at 5% and 10% (*w*/*w*) concentrations in the second experiment. (3) No differences in survival or immune responses were observed in both experiments. However, isopods exposed to TRWPs showed significant weight gain at lower concentrations but no gain at higher levels. Behavioural tests revealed increased vigilance in TRWP-exposed animals. Micro-FTIR analysis showed that the number of TMPs and TRWPs in the isopods correlated with soil concentrations, and particle size decreased during the experiment. (4) The study highlights the physiological and behavioural effects of TRWPs and the role of detritivorous species in the biofragmentation of TMPs and TRWPs, contributing to the biogeochemical plastic cycle.

## 1. Introduction

Tyre abrasion particles are one of the most important sources of pollution, and it is estimated that around 6,000,000 tonnes are produced worldwide each year [1,2,3]. Tyres consist of 40–50% natural rubber (polyisoprene) and synthetic rubber [styrene–butadiene rubber (SBR) or butadiene rubber]; 20–30% fillers such as soot/carbon black (C) silicon dioxide (SiO_2_), and chalk (CaCO_3_); 15% oils and resins; and a small percentage of preservatives and vulcanising agents [3,4,5,6]. In addition, tyre wear particles found in the environment are generated by the abrasion of tyres on the road surface [7] and are therefore rich in road-related compounds such as metals or organic chemicals (e.g., polycyclic aromatic hydrocarbons) that are potentially harmful to living organisms [8,9,10,11]. The highest concentrations of tyre particles were measured along roads with a range of 0.7 to 210 g/kg −1 t.w. [12], while in soil a range of 0 to 117 g/kg −1 t.w. was found, depending on the distance of sampling from the road and the location [13,14,15]. These particles are also present in water bodies from road runoff [16] and in PM10 and PM2.5 [17]. Nevertheless, fewer studies have been conducted on the potential effects on soil organisms [18,19]. Several ecotoxicological studies have looked at the effects of tyre particles in leachate on mainly aquatic organisms, showing toxic effects associated with metals and organic compounds in tyres [4,12,20]. Some recent studies have investigated the direct exposure of tyre particles to soil invertebrates such as woodlice, springtails, and earthworms [21,22,23,24,25]. Dolar et al. [22,23] investigated the effects of tyre particles on the immune system of the terrestrial isopod *Porcellio scaber* and found that there were no effects after short-term exposure, while immune-related genes in haemocytes and hepatopancreas were activated after 14 days of exposure. Other studies reported that ingestion of tyre particles by earthworms has effects on the gut microbiota, increases heavy metal levels, and provides the opportunity to transfer the particles to animals at higher levels of the trophic chain [21,25]. Terrestrial isopods are the only crustaceans that have succeeded in colonising the soil [26]. They can reach up to 1000 individuals/m^2^ of soil and often represent a dominant part of the soil fauna. They are distributed worldwide [27,28,29]. They are macro-decomposers and represent an important layer in the soil food web as they feed on decaying organic matter [30]. For this reason, they have been used in a large number of ecotoxicological studies [31]. Toxic effects on isopods can be monitored with different endpoints such as growth, survival, immune parameters, or behaviour [31]. The immune system of crustaceans is often sensitive to stressful environmental conditions, as evidenced by changes in haemocyte counts, prophenoloxidase (pPO) activation, phagocyte indices, and oxygen free radical release [32]. Consequently, the total haemocyte count (THC) and the activity of pPO-like enzymes are two common parameters used to assess immunological effects in crustacean experiments [33]. Microplastic consumption impairs cognitive functions, probably due to particle accumulation in the brain. In *Apis mellifera*, ingestion of a combination of plexiglass and polystyrene had a significant negative effect on sucrose responsiveness and impaired learning and memory formation in bees [34]. Imaging studies of the brains of the bees that had ingested these substances showed that microplastics penetrated their brains within just three days of oral ingestion and accumulated there, leading to learning disorders [35].

Our study aims to evaluate the effects of exposure to tyre microplastic particles (TMPs), i.e., tyre particles obtained under simulated laboratory conditions from worn tyres, and tyre and road wear particles (TRWPs), i.e., particles directly collected from the road surface. The side effects were assessed by analysing physiological parameters such as the growth, ingestion, and immune response of the terrestrial isopod *Armadillidium pallasii* (Brandt, 1833). In addition, the effects of TRWP exposure on the isopods were also investigated based on their behaviour and cognition. The focus was on two biologically relevant phenomena: the isopods’ phototaxis response and habituation. The phototaxis response can be measured by isopods’ preference for lighter or darker areas and is an ecologically relevant parameter, as it can influence the distribution of organisms in ecosystems. In general, isopods prefer dark areas in which to reside, but this preference may increase or decrease depending on their level of stress. For example, stressed killer crayfish (*Procambarus clarkii*) avoid entering dark areas where they would normally reside because they are more alert due to the presence of potential hidden threats and therefore adaptively switch their preference from dark to light locations [36]. Habituation, which is associated with both learning and response to stimuli, is related to an animal’s ability to adapt to redundant information in the environment. This involves reducing the response to repetitive, irrelevant stimuli so that animals can focus on biologically relevant tasks [37]. During habituation, animals learn about the properties of the stimuli, and their response gradually decreases. These two aspects, learning and responsivity, are crucial factors that influence an animal’s adaptation to its environment.

## 2. Materials and Methods

### 2.1. Characterisation of Microplastic Particles from Tyres (TMPs) and from Tyre and Road Wear Particles (TRWPs)

The TMP powder was obtained in the laboratory by dry abrasion of the tyre tread from two anonymised, dominant manufacturers. The tyres were worn to different degrees and varied in terms of production date: October 2003 and April 2015. The TMPs were then mixed with the test soil (1:1) using a vibrating mill for fine grinding (Mikro-Dismembrator, Braun Biotech, Melsungen, Germany).

The mixture of TRWP powder was collected in the town of Tychy in southern Poland on the national road No. 86 (50°09′54.9″ N 19°00′58.7″ E). TMP powder, a TMP–soil mixture, and TRWP powder were subjected to scanning electron microscopy (SEM) and energy-dispersive spectroscopy (EDS). The samples were mounted on aluminium stubs and coated with chromium (for TMP and TMP–soil mixture) and with carbon (for TRWP powder) using Quorum Q150T ES plus and analysed with Gemini300 SEM (Zeiss, Oberkochen, Germany) using secondary electrons at a working distance of 8.5 mm and an accelerating voltage of 20 kV. Energy-dispersive spectroscopy (EDS) was used for elemental analysis of the samples. EDS spectra and maps were recorded with an EDS probe XFlash 610 M (Bruker, Billerica, MA, US) with a recording time of 100 s and 250 s, respectively. The size of the particles was measured using the Fiji ImageJ software (version 2.9.0) [38].

### 2.2. Experimental Design

#### 2.2.1. Experimental Woodlouse

Specimens of *A. pallasii* were collected in 2022 from a suburban park in Gradisca d’Isonzo, Italy (45°53′28.5″ N, 13°30′03.9″ E). Before the experiment began, the organisms were acclimatised for four months at the University of Trieste under controlled conditions, such as a stable photoperiod of 12:12 (light/night) and a temperature of approximately 21.5 °C.

The animals were housed in glass enclosures (with a bottom surface area of 546 cm^2^) containing microwave-sterilised soil certified for organic farming, at a pH of 7 (Bioterril, Geotec, Cavanella Po, Italy). They were fed dry oak leaves and commercial crustacean food (Dr. Bassleer Biofischfutter, Aquarium Münster, Telgte, Germany) twice a week. Both organic soil and oak leaves were previously sterilised using a microwave (100 g of soil were irradiated for 1.5 min at 700 W and 100 g of leaves for one minute at the same power). The experiment utilised in total 120 healthy adult specimens of both sexes (average weight = 0.227 ± 0.05 g).

#### 2.2.2. Experimental TMP and TRWP Concentrations

Concentrations of 1.25%, 2.5%, 5%, and 10% (*w*/*w*) were tested in the TMP experiment, while 5% and 10% (*w*/*w*) were tested in the TRWP experiment. The concentrations were chosen based on Wik and Dave [12], who estimate the highest concentration of tyre particles in soil to be from 0.06 to 11.7%. To ensure comparable data on the soil conditions, its water potential was measured using a Dew Point Potentiometer (WP4C).

#### 2.2.3. Experimental Conditions

Five isopods were placed in each test glass terrarium (bottom area of 144 cm^2^). Three replicates were tested for each concentration and the control group. Then, a tyre–soil mixture (1:1) was prepared with a vibrating mill for fine grinding (Mikro-Dismembrator, Braun Biotech, Melsungen, Germany) at 2000× *g* per 30 s and used for the different concentrations in the TMP experiment, while the TRWP powder was sprinkled directly on the terrariums and mixed with the soil inside, as it was thinner and contained fewer aggregates. Before exposure, a layer of gypsum was placed on the bottom of the terrariums to increase surface friction and prevent the isopods from settling on the bottom. Each terrarium was filled with 19 g of soil and 1 g of leaves, 0.5 g whole and 0.5 g crushed. The isopods were weighed, and in the TRWP experiment a numbered label was attached to the back of the organisms with glue (Super Attack, “Loctite”). The isopods were exposed to a 12:12 (light/night) photoperiod for 30 days in an experimental room with an average temperature of 25.53 ± 0.89 °C (June–July 2022) in the TMP experiment and an average temperature of 18.8 ± 1.31 °C (January–February 2023) in the TRWP experiment.

### 2.3. Hemolymph Sampling

Before sampling, each woodlouse was placed in a tube and anaesthetised by placing the tube in ice for about 10 min. Using a sterile micropipette, the intersegmental membrane between the 5th and 6th tergite was punctured. The isopods were then gently squeezed, and the haemolymph was collected with a micropipette as described by Dolar et al. [24]. A drop was placed on a BLAUBRAND^®^ Bürker counting chamber (Electron Microscopy Science, Hatfield) to determine the total haemocyte count (THC), as described below. Approximately 10 μL of haemolymph was placed in a sterile Eppendorf tube (1.5 mL) and centrifuged at 13,000× *g* for 10 s, and the cell-free supernatant was immediately stored at 4 °C for analysis of pPO-like enzyme activity, as described below. After sampling, isopods were stored at −20 °C to analyse the content of tyre particles.

### 2.4. Stress Evaluation: Total Haemocyte Count (THC) and Prophenoloxidase-like Enzyme Activity

To perform THC, haemocytes were observed on the BLAUBRAND^®^ Bürker counting chamber under an Olympus BX50 microscope. Each sample was photographed at 10× magnification using a Lumix C-M4/3 camera (Panasonic, Kadoma, Osaka, Japan). Images were analysed using iji ImageJ software (version 2.9.0) with the Cell-Counter plugin, and THC was expressed as cell/mL. The pPO–like enzyme activity was measured by monitoring the formation of dopachrome v3,4-dihydroxyDL-phenylalanine (DL-DOPA, Sigma-Aldrich, Darmstadt, Germany) using a spectrophotometer (Infinite 200 PRO NanoQuant, Tecan, Männedorf, Switzerland), as reported by Giglio et al. [39]. A protocol described by Capanni et al. [40] and Manfrin et al. [41] was followed with some adaptations. Specifically, 4 μL of haemolymph was added to a sterile microtitre plate (Microtest Plate 96 Well, F, Sarstedt, Nümbrecht, Germany) placed over a cold pack. Then, 30 μL sodium dodecyl sulphate (SDS, 0.4 mg/mL in phosphate-buffered saline, AppliChem, Darmstadt, Germany) was added, and the mixture was incubated for 5 min at room temperature. SDS is known to be a chemical activator of PO from its inactive zymogen, prophenol oxidase (pPO) [42,43]. Then, 180 μL of DL-DOPA (3 mg/mL in phosphate-buffered saline) was added to the microplate and immediately recorded using a spectrophotometer (Infinite 200 PRO NanoQuant, Tecan, Männedorf, Switzerland). Enzyme activity was measured at 496 nm for 60 min at 2 min intervals and analysed as the slope (absorbance versus time) of the incremental activity and expressed as abs/μL/min.

### 2.5. Micro-FTIR Tyre Content Analysis

Tyre particles were analysed in the samples to determine both the total number of particles ingested and the particle size. Animals were digested with a solution of 20 mL of 30% H_2_O_2_ per gramme of tissue at 40 °C for 48 h under continuous mixing [44] under a fume hood with glove box (Iteco Engineering, mod. SGS20-13599) to prevent contamination of the samples with nanodust. The digested samples were filtered onto 0.45 μm filter fibre discs (Whatman) and sorted under a stereomicroscope (Nikon SMZ-800 N) to determine the total amount of particles per digested animal and their size (μm) using Nikon image analysis software (Nikon ACT-1). To ensure particle identification and exclude false positives, the collected particles were analysed using Fourier-Transform Infrared Microscopy (μFT-IR; Nicolet i-10 MX Infrared Imaging Microscope, Thermo Fisher Scientific, Waltham, MA, US). The device was equipped with a nitrogen-cooled detector MCT-A, which operates in the spectral range 7800–650 cm^−1^. The collected spectra were analysed with the Thermo Scientific OMNIC Picta user interface. The spectra of tyre dust were collected using the μFT-IR technique on both original dusts and dusts collected in the field. Nearly ten replicates were collected to calculate the mean spectrum, which was added to the spectral library as a reference for comparison with the target particles.

### 2.6. Behavioural and Cognitive Tests

**Figure 1:**
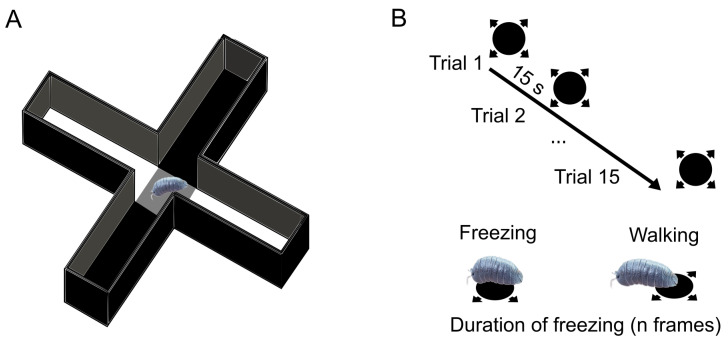
(**A**) The plus maze used for the phototaxis test. (**B**) The sequence of stimulus repetitions during the habituation test and freezing response of the isopods.

Anxiety and habituation tests were performed on the first and last day of exposure to TRWPs. For the anxiety level test, the experimental set-up consisted of a 3D-printed black “plus maze” made of PLA^®^ to ensure uniformity of the wall (Figure 1A). Each maze branch was 10 cm long, 1.5 cm wide, and 5 cm high. The centre of the maze was separated from the branches by removable doors. The maze was in the centre of a flat LCD monitor (1920 × 1080 pixels, 29 fps), on which a 2D sketch of the maze was projected. In this sketch, two of the branches on opposite sides of the maze were filled in black (RGB: 0, 0, 0), and two were filled in white (RGB: 255, 255, 255), while the central square of the maze was filled in grey (RGB: 127, 127, 127). At the beginning of the experiment, the isopods were placed in the centre of the maze for one minute before the doors were opened manually by the experimenter. The isopods’ exploration of the maze was then recorded from above with a camera for 10 min. The recorded videos were analysed using a custom-made tracker. We assessed the probability that a woodlouse first entered a black or a white branch and the time it spent in each branch. For the habituation test, a circular arena (diameter = 20 cm, height = 40 cm) was set up above a flat LCD monitor (1920 × 1080 pixels, 29 fps). The choice of a circular arena for this test was due to the fact that isopods tend to be more active in circular environments than in those with corners where they can linger. A custom-made programme built using PsychoPy 2023.1.2 [45] projected the habituation stimulus into the centre of a white screen on command from the experimenter. The habituation stimulus consisted of a looming black disc (RGB: 0,0,0) with an initial visual angle size of 0.5° that expanded with each frame according to the formula θ(t) = 2 tan−1 (L/vt), where θ is the magnification of the disc, *t* is the frame number from the stimulus onset, l is the radius, and v is the rate of expansion. The expansion took place over 29 frames (1000 ms). The disc then remained projected at its maximum extent for a further 15 frames (517.5 ms), covering the entire floor of the arena. The habituation test consisted of 15 repetitions (trials) of the threatening stimulus with an interval of 15 s between repetition (Figure 1B). If the animal was immobile when the stimulus was scheduled to appear, the trial was delayed up to a maximum of 30 s. The test was recorded from above with a camera, and the video was analysed offline using a specially developed tracking script [based on Python and using OpenCv 4.10.0 [46]. Specifically, we analysed the duration of the animals’ freezing (number of frames the animals remain motionless) in each trial.

### 2.7. Statistical Analysis

All statistical analyses were performed using R software version 4.0.5 [47]. Of the measured variables—weight, THC, pPO-like activity, and particle size—only weight in the TMP experiment and THC in the TRWP experiment were normally distributed after performing a Shapiro–Wilk test. The homogeneity of variance of all variables was confirmed by an F-test for normal variables and the Fligner–Killeen test for non–normal variables, while for THC, size, and pPO-like activity, the Levene test was used. Weight differences before and after the 30-day exposure period were compared using the paired *t*-test or the Wilcoxon test. In the TMP experiment, some observations were randomly cancelled to obtain the same number of animals before and after exposure required to perform the paired tests. After random deletion, a *t*-test was performed to check if there were differences within each replicate, and no differences were found. This procedure was necessary because the isopods were not labelled. Particle size and THC mean difference between tested groups were compared using the Kruskal–Wallis test or ANOVA. For particle size, Dunn’s pairwise post hoc comparisons with Bonferroni correction were performed. For the analysis of pPOlike activity, a linear model was used to calculate the incremental activity of the enzyme. The incremental slopes between tyre concentrations were compared using the Kruskal–Wallis test. Boxplots were drawn using functions of the R package “ggplot2” [48]. Kaplan–Maier curves at constant risk were calculated with the R package “survival” [49] and plotted with the R package “survminer” [50], while a log-rank test was performed to test the significance of the curve differences.

Data from the phototaxis and habituation tests were analysed using linear mixed-effects models with TRWP concentration (control, 5% and 10%) and the test day (“pre-exposure” and “post-exposure”) as factors. To fit the linear models, we used the “lmerTest” package [51]. All differences were considered significant when the *p*-value was ≤0.05. All results are presented as mean ± standard deviation unless otherwise stated.

## 3. Results

### 3.1. Characterisation of the Particles

**Figure 2:**
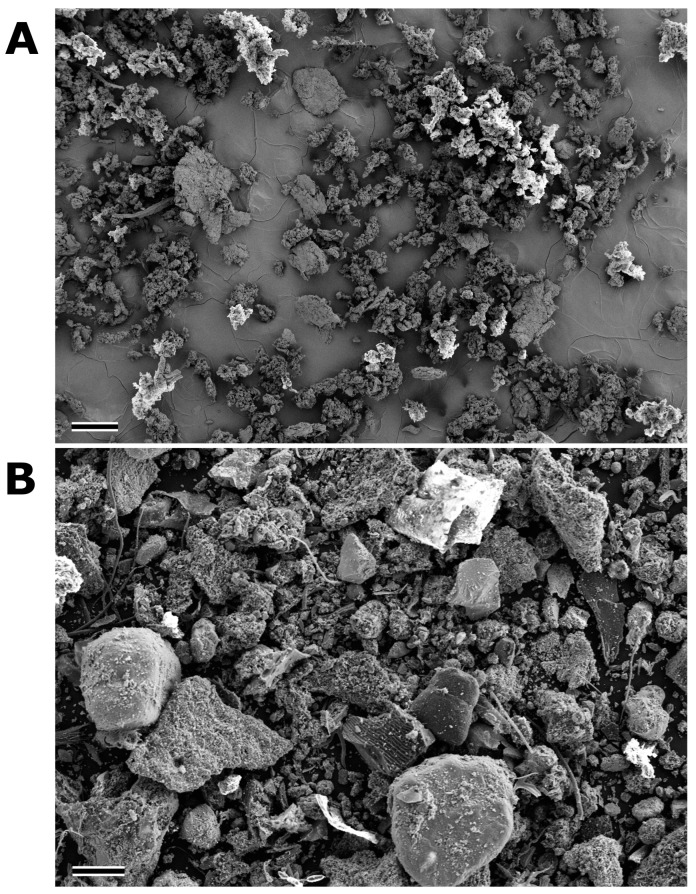
(**A**) Scanning electron microscope image of the tyre–soil mixture. (**B**) Scanning electron microscope image of TWRP powder showing the large heterogeneity in particle size and shape. Scale bar = 200 μm.

The SEM image of the tyre–soil mixture shows the presence of aggregates and heterogeneity in particle size (Figure 2A), ranging from 15.4 μm to 422.6 μm. EDS analysis of the TMP powder (Table 1) revealed that mainly carbon (57.21–62.28 mass per cent) and oxygen (22.70 to 23.73 mass per cent) were present, while about 7.69 to 11.60 mass percent silicon was present. Sulphur was present in traces, while zinc was only found in part of the sample. The presence of chromium was due to the coating of the sample with this element. The same elements were also present in the tyre soil sample; additionally, calcium, iron, and aluminium were present in traces and were attributed to the soil component (Table 1).

Figure 2B shows an SEM image of the TRWP sample and reveals a large difference in particle size and shape. The size of the TRWPs ranges from 1.9 μm to 870.4 μm. This heterogeneity is also reflected in the chemical composition (Table 1) with the largest proportion of carbon (47.41 to 57.32 per cent by mass) and oxygen (35.10 to 37.14 percent by mass) and the presence of around 3.34 to 6.12 percent by mass silicon. Compared to the mixture of tyre powder and tyre soil, no traces of zinc were found, while potassium, sodium, and magnesium were present in traces. Titanium was only found in traces and is not widespread in the sample area.

### 3.2. Survival

**Figure 3:**
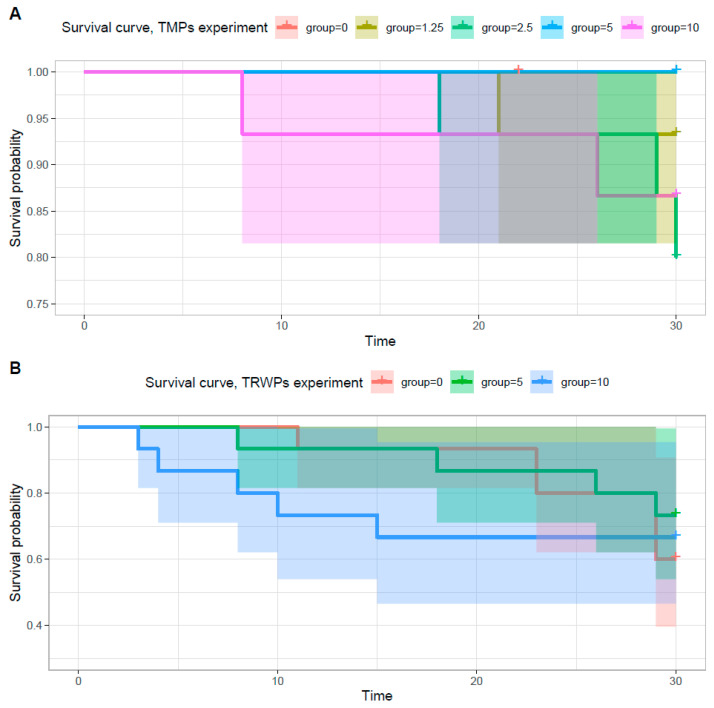
(**A**) TMP experiment: Kaplan–Meier distribution of the survival of the exposed and the control groups. The diagram shows the probability of survival and deaths during the exposure period. “*p*” refers to the *p*-value of the long–rank test. The time is given in days. (**B**) TRWP experiment: Kaplan–Meier survival distribution of the exposed and the control groups. The diagram shows the probability of survival and deaths during the exposure period. “*p*” refers to the *p*-value of the log-rank test. The time is given in days.

After 30 days of exposure, 67 of 75 individuals survived in the TMP experiment, while 29 of 45 individuals survived in the TRWP experiment. The Kaplan–Meier constant risk curves of the TMP experiment (Figure 3A) showed that the probability of survival was 100% in the control group, 93% in the 1.25% (*w*/*w*) group, 80% in the 2.5% (*w*/*w*) group, 100% in the 5% (*w*/*w*) group, and 87% in the 10% (*w*/*w*) group.

In the TRWP experiment, the survival probability (Figure 3B) was 60% in the control group, 73% in the 5% (*w*/*w*) group, and 67% in the 10% (*w*/*w*) group. In both experiments, no difference in survival probability was observed between the control group and the exposed group.

### 3.3. Growth

**Figure 4:**
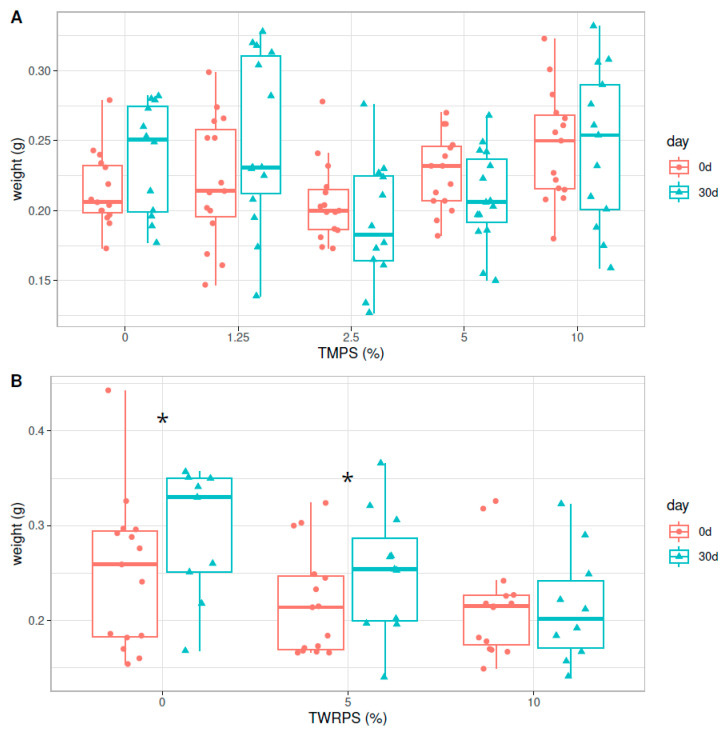
Boxplots of the average weight of the exposed and control group before and after 30 days of exposure in the TMP (**A**) and TRWP experiment (**B**). Significant differences between 0 and 30 days after exposure are marked with an asterisk (*).

In the TMP experiment, no significant weight differences were observed before and after 30 days of exposure (Figure 4A), whereas in the TRWP experiment (Figure 4B), a significant increase of 0.044 g was observed in the control group and of 0.025 g in the 5% (*w*/*w*) group (paired Wilcoxon test, *p*-value = 0.04, *p*-value = 0.05), while the 10% (*w*/*w*) group did not gain weight.

### 3.4. Immunological Parameters

**Figure 5:**
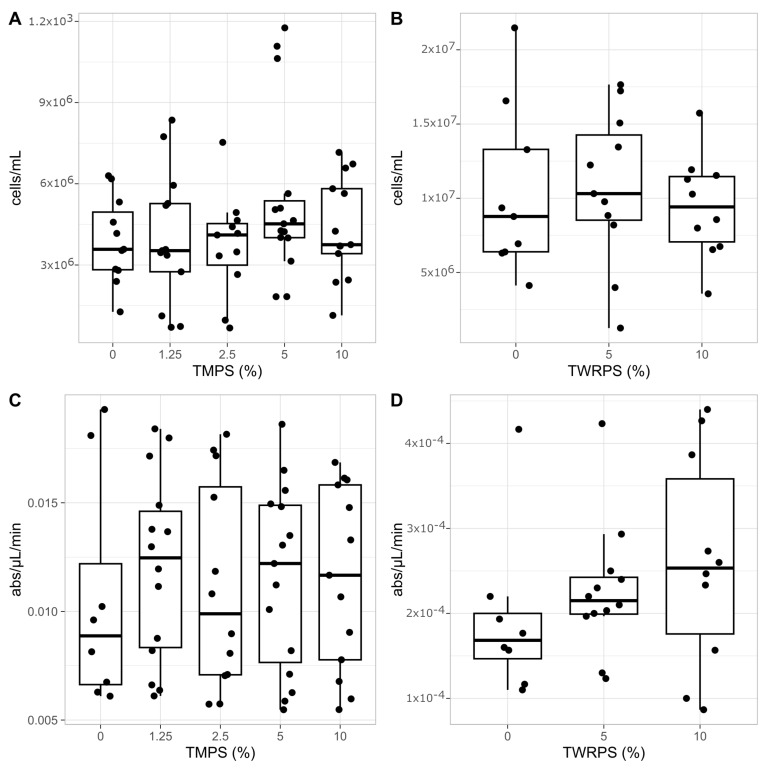
Boxplot of THC of the exposed group and the control groups in the TMP (**A**) and TRWP experiment (**B**). Boxplot of the pro-phenoloxidase activity of the exposed and control groups in the TMP (**C**) and TRWP (**D**) experiments.

High variability in THC levels was observed in each group, and in both experiments the difference between the exposed and control groups was not significant (Figure 5A,B). The same result was observed for pPO-like activity, although a variable trend was observed between the tested groups in the TMP experiment and an increasing trend in the TRWP experiment (Figure 5C,D). In the TRWP experiment, the lowest value was observed in the control group (3.12 × 10^−5^ ± 1.78 × 10^−5^ abs/μL/min), and the highest value was observed in the 10% (*w*/*w*) group (4.10 × 10^−5^ ± 2.16 × 10^−5^ abs/μL/min).

### 3.5. Content of the Tyre Particles

**Figure 6:**
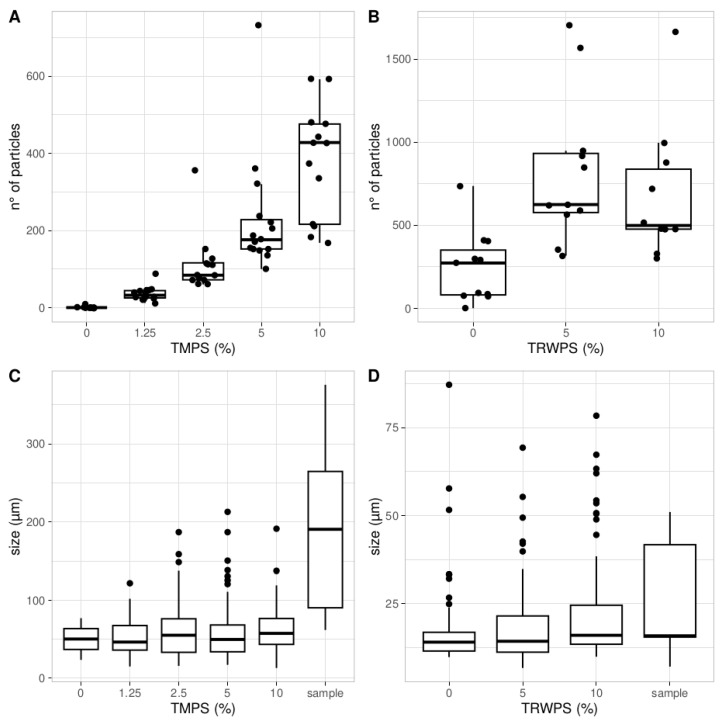
Boxplot of the average number of particles found in the isopods in the TMP (**A**) and TRWP (**B**) experiments. The presence of particles in the control groups indicates cross-contamination, especially in the TRWP experiments, indicating the high volatility of this compound. Boxplot of size of the particles found in the isopods in TMP (**C**) and TRWP (**D**) experiments; the boxplot labelled “sample” indicates the size of the tyre particles measured in the samples of TMPs and TRWPs used for the experiment.

Micro-FTIR analysis was performed to determine the number and size of particles in the isopods after exposure. In the TMP experiment (Figure 6A), the number of particles increased with increasing tyre concentration on the soil, with the lowest value recorded in the 1.25% group (36.0 ± 18.6) and the highest recorded in the 10% group (379.1 ± 146.7). The size of the tyre particles (Figure 6C) was smaller (overall mean of 55.8 ± 15.9 μm) than the particle size in the tyre–oil mixture used in the experiment (137.8 ± 49.9 μm), and the difference between the exposed groups and the sample was significant (Kruskal–Wallis, *p* < 0.001). In the TRWP experiment, the highest number of particles was found in the 5% group (822.5 ± 451.5), while the number in the 10% group was 683.2 ± 411.8 (Figure 6B). Some particles were found in the control group (248.4 ± 215.9), indicating the high volatility of the TRWP powder. The size of the particles (Figure 6D) increased with increasing TRWP content and was smaller (18.9 ± 12 μm) than the particles in the TRWP powder (24.6 ± 18.1 μm) used in the experiment, but this difference was not significant.

### 3.6. Results of Behavioural and Cognitive Tests

**Figure 7:**
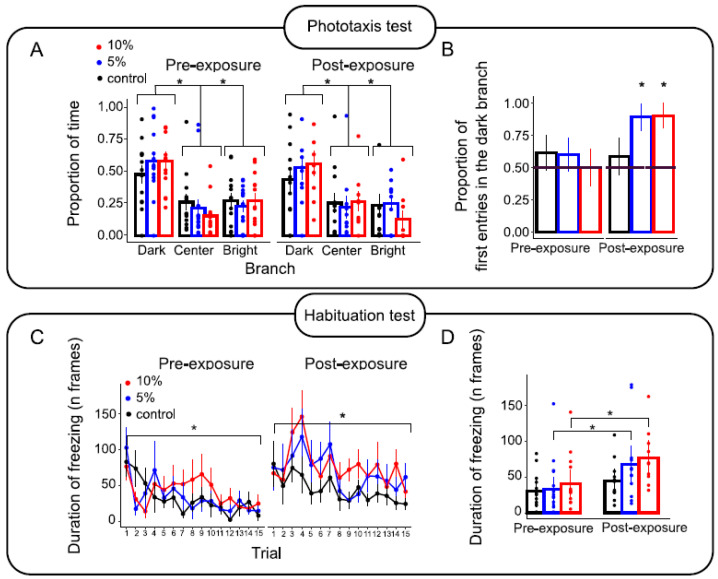
(**A**) The proportion of time spent in dark and light branches and the time spent in the centre of the plus maze (grey area, see panel A of Figure 1). (**B**) The proportion of first entries into the dark branch under different conditions; the dashed line represents the random level. (**C**) The average freezing time after trials and trial days under different concentration conditions. Panel (**D**) shows the same freezing times averaged over all trials. In all the panels, the error bars represent ± 1 s.e.m. The asterisks indicate *p* < 0.05.

Test of phototaxis: The results are shown in Figure 7A. The analysis showed that, on average, the isopods spent more time in dark branches than in light branches in the maze (*t*(132) = 7.18, *p* < 0.001), indicating a baseline preference for darkness known as negative phototaxis. Before exposure, the likelihood of isopods entering a dark branch first was at chance level in all conditions; however, after exposure, this probability increased significantly for isopods in the 5% and 10% conditions (5% proportion of first entries in a dark branch: 0.88, z = 2.20, *p* = 0.027; 10% proportion of first entries into a dark branch: 0.90, z = 2.37, *p* = 0.017), while it remained at chance level for the isopods in the control group (proportion of first entries into a dark branch: 0.58, z = 0.57, *p* = 0.564). This result indicates that the isopods were more likely to enter dark areas first after TRWP exposure when placed in an unfamiliar environment.

Habituation test: The results are shown in Figure 7B. From the initial pool of 1154 responses, we excluded 47 outliers (approximately 4% of the total data) that were over ±2.5 standard deviations from the linear model estimates. The analysis revealed a main effect of stimulus repetition (F(14, 91.09) = 2.57, *p* = 0.003), test day (F(1, 647.99) = 46.83, *p* < 0.001) and a two-way interaction between stimulus repetition and test day (F(14, 574.89) = 2.90, *p* < 0.001) and between concentration condition and test day (F(2, 563.79) = 4.63, *p* = 0.010). In particular, habituation was observed on the “pre-exposure” test day, which was reflected in a significant decrease in freezing duration triggered by the looming stimulus from trial 1 to 15 (t(102.57) = −5.090, *p* < 0.001); the same decrease continued “post-exposure”, albeit less pronounced (“post-exposure”: t(132.45) = −2.00, *p* = 0.046) (Figure 7C). This difference was probably due to the 5% and 10% concentration conditions, as the isopods in these two groups froze significantly longer after exposure than before exposure (for the 5% condition: t(359) = −5.60, *p* < 0.001; for the 10% condition: t(291) = 3.95, *p* < 0.001). By contrast, the isopods in the control group froze for a similar length of time on both test days (t(244) = 1.52, *p* = 0.129). The results show that TRWP exposure increased isopods’ responsivity to the looming stimulus.

## 4. Discussion

In both experiments, no statistical differences in survival probability were observed, consistent with similar studies on *Porcelio scaber* exposed to tyre particles at concentrations up to 1.5% *w*/*w* for three weeks [23,24]. It is likely that exposure to either powder had no impact on the survival rate of terrestrial isopods during the 30-day exposure period. Although survival probability was lower in the TWRP experiments compared to the TMP experiments, this discrepancy may be due to external factors such as the labelling method, which might have hindered moulting, or seasonal differences, as the TMP experiments were conducted in winter and the TWRP experiments were conducted in summer.

Growth was assessed by measuring weight. After 30 days of exposure to TMPs, no significant weight changes were observed, a finding consistent with similar studies by Solenen et al. [24] and Dolar et al. [23], which also reported no significant differences in the feeding activity of *P. scaber* following three weeks of exposure to tyre particles. However, the TWRP experiments yielded different results; a significant weight increase was observed in both the control group and the group exposed to 5% (*w*/*w*) TWRPs, while no significant decrease was seen in the group exposed to 10% (*w*/*w*) TWRPs. This suggests that higher concentrations of TWRPs affected weight gain over the 30-day exposure period. Immune markers are critical indicators of stress in crustaceans [32], with THC and pPO activity frequently used in related experiments [23,,52]. In our experiments, no significant differences in immune parameters were detected between the exposed and control groups, and THC levels showed considerable variability. This variability is likely due to individual physiological factors such as moulting, starvation, and diet composition [53,54], which have also been linked to the wide range of THC levels reported in previous studies [23,24,55]. In the TWRP experiment, pPO-like activity showed an increasing trend with higher doses, although the differences between the exposed and control groups were not statistically significant. Phenoloxidase, a key enzyme in invertebrates, plays a central role in melanogenesis and tissue repair and is also involved in processes such as microbial agglutination and killing [33,56,57,58]. Due to the complexity of the PO system, interpreting the pPO trend can be challenging, but, generally, elevated levels are associated with immune activation and stress responses [24,58].

The elevated pPO levels and lack of growth in the 10% groups suggest that high concentrations of TRWPs may induce a stress response in the isopods. However, no changes in pPO levels were observed in *P. scaber* exposed to up to 1.5% tyre particles for three weeks [24], and a decrease in pPO levels was found in crayfish after 21 days of exposure to PE microplastics [59]. Therefore, further studies are needed to investigate this enzyme’s response more comprehensively.

One of the most noteworthy findings is the micro-FTIR spectroscopy analysis of particle content in the isopods. In both experiments, particles were detected inside the isopods, confirming their uptake. Additionally, in the TMP experiment, a dose-dependent increase in particle quantity suggests that accumulation occurred inside the isopods, potentially leading to digestive system blockages. Although whole animals were digested during the micro-FTIR analysis, the size of the particles (average size of 55.8 ± 15.9 μm) suggests they were trapped in the digestive system, as particles of this size are unlikely to migrate to other tissues. Previous studies have reported TMP uptake in *Enchytraeus crypticus*, along with alterations to its gut microbiota [21]. Similar findings were observed in *Eisenia fetida* at a lower concentration of tyre particles (1% *w*/*w*) [25], with the authors suggesting the potential transfer of particles to chickens. Like woodlice, earthworms occupy lower trophic levels and can ingest particles that may be passed along the food web. The significantly smaller particles found inside the isopods (55.8 ± 15.9 μm) closely match the sizes of that in the tyre–soil mixture used in the experiments (137.8 ± 49.9 μm), indicating substantial fragmentation. This highlights the ecological role of terrestrial isopods as macrodecomposers, particularly in breaking down materials [30]. Similar fragmentation has been observed in amphipods (*Orchestia gammarellus*) and earthworms (*Eisenia andrei*) in studies on microplastics [60,61], suggesting that these organisms may contribute to the formation and spread of microplastics in the environment.

In the TWRP experiment, no dose dependency was observed, and some particles were also found in the control samples. This could be attributed to the variability in particle size and shape detected in SEM and EDS analyses, as well as the high volatility of the TRWP mixture. Nevertheless, as with TMPs, smaller particles were found in the isopods than those used in the TWRP experiment, although the difference was not statistically significant. Due to the sublethal effects observed in the TMP experiment, behavioural and cognitive tests were conducted in the second experiment (TWRPs).

No dose-dependent trend was observed in the TWRP experiment, and some particles were also found in the control samples. This could be related to the large variations in size and shape of the TRWP powder detected in SEM and EDS analyses, as well as to the high volatility of the mixture. Nevertheless, as with the TMPs, smaller particles were found in the isopods than the particles of TRWP powder used in the experiment, although this difference was not significant. In view of the sublethal effects resulting from the TMP experiment, behavioural and cognition tests were carried out in the second experiment (TWRP experiment).

The behavioural patterns of isopods exposed to 5% (*w*/*w*) and 10% (*w*/*w*) TRWPs were altered compared to isopods in the control conditions. It was only after these treatments that isopods exhibited an overall heightened reaction to the irrelevant stimulus, indicating an increased alertness that was further confirmed by the post-treatment higher negative phototaxis. The shift towards preferring dark areas suggests that treated isopods were more inclined to find dark spots where they could camouflage themselves with the background instead of bright areas where they would be more visible. This behaviour suggests that ingestion of microplastics prompted isopods to increase their vigilance in response to stressful situations. Nevertheless, isopods’ learning abilities as measured by the habituation curve remained unaltered, thus showing a specificity of the effect of the treatment in heightened alertness across distinct tasks. It is important to emphasise that our evaluation of habituation allows us to distinguish between true habituation and mere fatigue. This is reflected in the decrease in the freezing response associated with an increase in locomotor activity, which is in no way synonymous with fatigue. To summarise, neuronal functionality appears to be somewhat altered by TRWP exposure, even at low concentrations and after only 30 days of treatment. It is possible that the TRWPs ingested by isopods are also transported to their brain via the haemolymph and influence their behaviour, information processing, and learning [35]. So far, however, this remains a hypothesis, as there is a possibility that the treated environment is perceived as altered (possibly through an unpleasant taste or odour). It is difficult to distinguish between a general feeling of sickness and increased alertness triggered by the presence of an irritating substance, which could be investigated in future studies.

## 5. Conclusions

This study shows that high concentrations of TRWPs can affect the growth and behaviour of the terrestrial isopod *A. pallasii*. Our results show that both tested particles (TMPs and TRWPs) are ingested and fragmented by woodlice. This process could affect the food web and the environment and increase the dispersal of these particles. A detailed analysis of faecal pellets and the comprehensive characterization of TRWPs (a complex mixture) will refine and enhance the findings from these experiments. This study provides important insights into the effects of two compounds widely used in the environment [2] on terrestrial isopods, which are not yet very well studied, especially with regard to TRWPs.

For this reason, further studies are needed to better understand the potential impacts and hazard implications of these particles.

## Figures and Tables

**Table 1:** Percentage mass of elements in the samples analysed by energy-dispersive spectroscopy (EDS).

Element	Mass (%) in TMPs	Mass (%) in TMPs–Soil	Mass (%) in TRWPs
Carbon	57.51–62.28	59.28	47.41–57.32
Oxygen	22.70–23.73	27.26	35.10–37.14
Silicon	7.69–11.60	7.56	3.34–6.12
Chromium	5.98–6.37	3.59	-
Sulphur	0.96–1.49	0.77	0–0.19
Calcium	-	0.71	1.69–3.92
Iron	-	0.30	0.63–2.08
Aluminium	-	0.27	0.47–1.90
Zinc	0–0.59	0.27	-
Potassium	-	-	0.33–0.65
Sodium	-	-	0.33–0.34
Magnesium	-	-	0.22–0.38
Titanium	-	-	0–0.61

## Data Availability

Dataset available on request from the authors.
